# Biomechanics of the Hammer Throw: Narrative Review

**DOI:** 10.3389/fspor.2022.853536

**Published:** 2022-03-31

**Authors:** Gian Mario Castaldi, Riccardo Borzuola, Valentina Camomilla, Elena Bergamini, Giuseppe Vannozzi, Andrea Macaluso

**Affiliations:** Laboratory of Bioengineering and Neuromechanics, Department of Movement, Human and Health Sciences, University of Rome “Foro Italico”, Rome, Italy

**Keywords:** hammer throw, athletics, biomechanics, throws, performance, track and fields, technique

## Abstract

Hammer throw is a discipline characterized by unique biomechanical features, which have often captured the interest of scientists and coaches in athletics. However, most studies have been published on technical journals for coaches and there are only few works on the biomechanical aspects of hammer throw in scientific literature. This narrative review provides a critical evaluation of the articles published in scientific and the most relevant technical journals with a particular focus on the biomechanical aspects that underlie the throwing technique and contribute to performance enhancement. The modern throwing technique has many elements in common with that used by the best throwers in the Eighties, underlying a limited development in the biomechanical understanding of throwing motion in recent years. This review analyses the ballistic and environmental aspects of the discipline as well as the motion of the center of mass of both the hammer and thrower. Furthermore, the orbital movement of the hammer and the forces involved in the throw are evaluated. This review emphasizes the kinematic and dynamic parameters that emerge as the most relevant to improve the throwing performance. Among these, linear release velocity appears to be a fundamental element. To maximize this variable, the athlete is required to accelerate the hammer by applying force. The curve of the time-tangential velocity of the hammer follows a trajectory very similar to that of the forces applied to the hammer-thrower system indicating a strong relationship between the two variables. The thrower uses the action of the leg muscles to gain momentum, which is then transferred to the hammer through the trunk and arm muscles, thus obtaining an increase of the linear release velocity. This review provides coaches with a critical analysis of the hammer throw technique, highlighting relevant factors for future development of training programmes. Our work reveals a substantial gap in the literature, particularly concerning the evaluation of fundamental key aspects of the throw such as the assessment of preliminary winds, the entry to the first turn and the definition of the rotation axes involved in the throw. A more in-depth analysis of these key elements is required to improve the understanding of the biomechanics of hammer throw.

## Introduction

Hammer throw is a relatively recent discipline when compared to other disciplines in athletics, many of which already existed in Ancient Olympic Games, such as running competitions or discus throwing. The origins date back to the Highland Games, in which a real blacksmith's hammer was thrown (Quercetani, 2008). Hammer throw has been part of the Modern Olympic Games since 1900 for men and since 2000 for women. The discipline involves throwing with both hands as far as possible an implement consisting of a metal ball, a steel cable and a metal handle from a circular platform 2.135 m long and 34.92 degree wide (in sexagesimal degrees, with its origin in the center of the platform). For men, the hammer has a minimum mass of 7.260 kg, the cable a maximum length of 121.5 cm and the sphere a diameter between 11 cm and 13 cm. For women, the mass is 4 kg minimum, the length 119.5 cm maximum and the diameter between 9.5 cm and 11 cm. The game rules are governed by the World Federation and have been the same all over the world for about 15 years, even at youth level. The men's world record is 86.74 m (Yuri Sedykh, Stuttgart, 30/8/1986) and the women's one is 82.98 m (Anita Włodarczyk, Warsaw, 28/8/2016).

**Figure 1:**
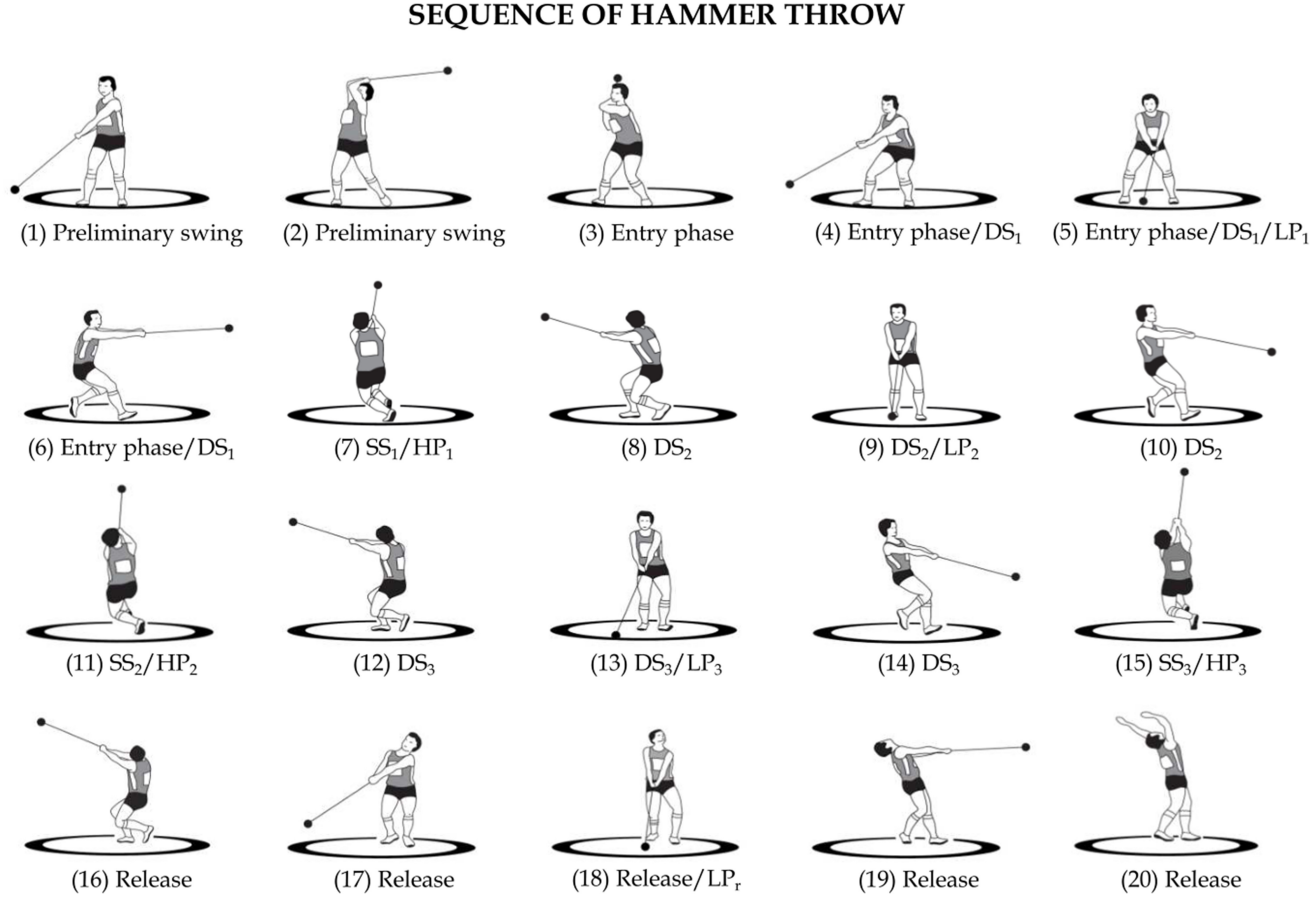
Phases of Hammer Throw. Preliminary swings [1–3]. Entry phase [3–4–5–6]. Single support of the first turn (SS_1_) [6–8]. Double support of the second turn (DS_2_) [8–10]. Single support of the second turn (SS_2_) [10–12]. Double support of the third turn (DS_3_) [12–14]. Single support of the third turn (SS_3_) [14–16]. Release [16–20]. First turn (T_1_) [3–8]. Second turn (T_2_) [8–12]. Third turn (T_3_) [12–16]. Low point of the hammer in the first turn (LP_1_) [5]. Low point of the hammer in the second turn (LP_2_) [9]. Low point of the hammer in the third turn (LP_3_) [13]. Low point at release (LP_R_) [18]. High point of the hammer in the first turn (HP_1_) [7]. High point of the hammer in the second turn (HP_2_) [11]. High point of the hammer in the third turn (HP_3_) [15].

The most commonly used throwing technique (Figure 1) consists of a series of preliminary swings followed by a series of turns and ends with the release of the implement (Bondarchuck, 1987; Burke et al., 1989; Gaede, 1990). In the preliminary swings, which are usually two or three, the thrower stands at the rear of the circle of throw, facing the opposite direction of the throw and, then, rotates the hammer over the head counter clockwise or clockwise, for right-handed or left-handed athletes, respectively. For the sake of clarity, henceforth, the throwing technique of a right-handed thrower will be considered. These rotations are used to overcome the inertia of the hammer, which is initially stationary, place it on an orbit and give it a suitable velocity to begin the entry phase in which the thrower begins the turns (Dapena, 1986; Dapena and Feltner, 1989). Thus, the thrower makes three or four turns on himself (which can rarely be five) while holding the hammer with both hands. During the turns, the thrower performs a combined movement of rotation and translation (Susanka, 1986; Murofushi et al., 2005). In fact, the left foot is always in contact with the circle of throw across which it translates with a series of shifts from heel to toe. This way, the thrower passes from the rear edge of the circle of throw to the front edge, from which the distance traveled by the hammer, i.e., the throw performance, is measured (Ohta et al., 2010; Brice et al., 2011). Simultaneously, the right foot alternates between phases in which it is in contact with the circle of throw and phases in which it is raised.

**Figure 2:**
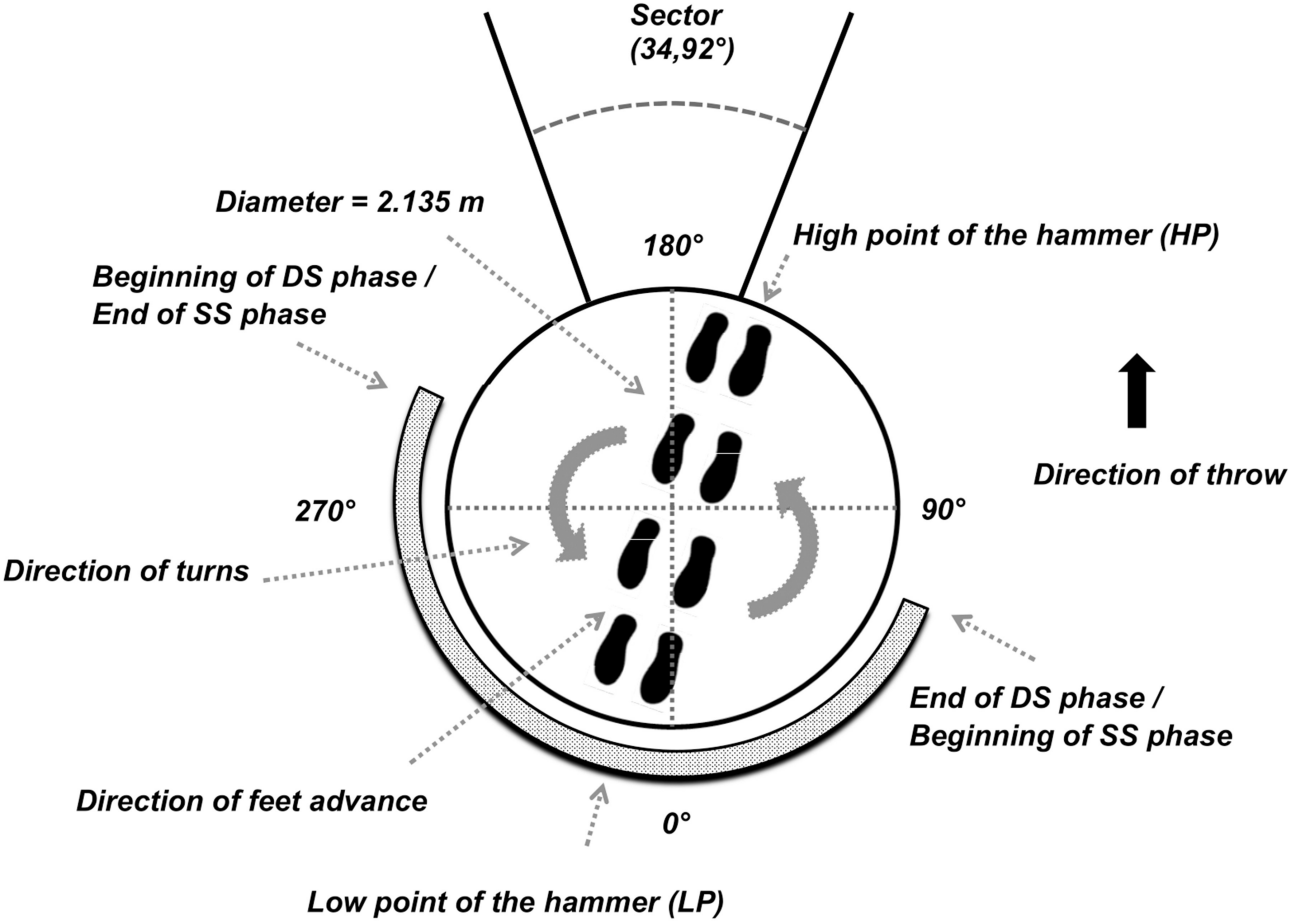
Azimuthal displacement of high point of the hammer (HP), low point of the hammer (LP), SS and DS phase.

Given the substantial rotational characteristics of the throw, the technical literature usually refers to “azimuthal angles” (Samozvetov, 1974) defined in the horizontal plane to better describe this complex motor task. Thus, an azimuthal angle provides the position of the hammer with respect to the center of mass of the hammer-thrower system in an overhead view. The time-behavior of this angle is generally used to identify specific events within a turn (Figure 2). In each turn, it is therefore possible to distinguish two phases of about half a turn: a double support phase (DS) and a single support phase (SS). During the DS phase, the hammer reaches its lowest point (LP) and during the SS phase its highest point (HP). During the turns, the DS phase usually begins with azimuthal angles of 230°- 270° and ends with azimuthal angles of 40°-90° depending on the technique of the thrower (Bondarchuck, 1987) (Figure 2). The last phase of the throw is the release, in which the thrower is positioned with both feet on the circle of throw and extends the legs and hips with a simultaneous rotation of the upper body just before releasing the implement at about 90° azimuth (Figure 2) (Dapena, 1984, 1986; Susanka, 1986; Dapena and Feltner, 1989; Dapena and Mc Donald, 1989; Murofushi et al., 2005; Ohta et al., 2010; Brice et al., 2011).

This review will address ballistic and environmental aspects of hammer throw as well as the motion of the center of mass of both the hammer and thrower. Kinematics and kinetics of the hammer, the thrower and hammer-thrower system will be evaluated with emphasis on the main factors contributing to throwing performance.

## Ballistic and Environmental Aspects

From the instant at which the hammer is released, it follows the motion of a projectile, whose motion equation is known under the assumption that the effects of air resistance are negligible. Unlike other athletics disciplines, such as discus or javelin, in which aerodynamics plays a fundamental role, in hammer throw the difference in shape and length of the hammer trajectory in real environmental conditions, which will be analyzed in the next paragraphs, are much smaller compared to a hypothetical throw into the void. In the absence of air, the hammer follows a parabolic motion after it is released, the length of which depends on release height, release angle and release velocity (Pozzo, 1992). The trajectory of the hammer center of mass consists of a parabola, the range of which can be determined by the following equation:


(1) $$\begin{array}{l} L=v_{0}^{2}cos\varphi \frac{sin\theta +\sqrt{\sin^{2}\varphi +\frac{2gh_{0}}{v_{0}^{2}}}}{g} \end{array}$$

in which, *v*_0_ is the velocity of the hammer at the instant of its release, *g* is the acceleration due to gravity, *h*_0_ the height at the instant of its release and φ the angle of release with respect to the horizontal. From a mathematical point of view, the equation indicates that the range increases as the hammer height and velocity increase at release, while the optimal release angle changes as the other two variables change (Pozzo, 1992; Brice et al., 2018).

Some additional considerations should be made regarding the throwing motion. If the thrower's goal is to maximize the length of the throw, he/she must find the optimal combination of the three variables (speed, angle, and height of release) (Otto, 1990; Dapena et al., 2003). With the current throwing technique, even considering all the subjective technical interpretations, the release height is strongly conditioned by the anthropometric characteristics of the thrower and is approximately equal to the height of the shoulders from the ground. Bartonietz (2008) pointed out that seeking a release height greater than that of the shoulders, although hypothetically profitable, would result in an excessive decrease in the release velocity due to the direction of the force vector which slows the hammer, with a consequent shortening of the throw length.

Given a release height of approximately that of the shoulders, the optimal release angle is slightly <45°. In particular, for throws between 60 to 80 m the optimal release angle would lay around 44°. In practice, it has been observed that athletes tend to throw with a lower release angle, from 40° to 42°, with a certain variability from athlete to athlete (Pavlović, 2020a,b). The difference between the optimal angle and the observed angle in the world's best throwers is to be attributed to a technical compromise. Indeed, the search for an optimal angle from a mathematical point of view would determine an excessive decrease in the release speed, thus reducing the length of the throw (Bartonietz, 2008). Therefore, once the thrower has built a technique that optimizes release height and release angle, the only variable that he/she can work to increase the throw length is the release speed (Bondarchuck, 1987; Morris and Bartlett, 1991).

Several studies have been carried out on the influence of environmental and ballistic factors in hammer throw. These studies report that, for throws around 75 m, the presence of air decreases the range by about 3%, while for every 2 ms^−1^ of tailwind the increase in length of the throw is about 60 cm. The direct or indirect influence of gravity, centrifugal force and Coriolis forces at different points on the Earth was also investigated (Jánosi and Bántay, 2002). For an altitude difference of 1,000 m, the distance traveled by the hammer may change up to ± 55 cm, whereas considering latitude, a change of about ± 45 cm was found between the pole and the equator, which corresponds to a change of 34 cm between latitudes of 67,5° and 17,5°, i.e., the extremes of the planet within which competitions usually take place. Temperature fluctuations of ± 10°C lead to variations in the throw length of ± 17 cm (Mizera and Horvath, 2002). With respect to air pressure, the throw performance varies by about ± 8 cm every ± 2 kPa (P_0_ = 101 325 kPa) and the Coriolis force throwing from East to West, when opposed to a throw in the opposite direction (180°), returns a difference of 3.4 cm to which 1.5 cm are added depending on whether an athlete throws on one edge or the other of the throwing sector (40°). Finally, variations in the inclination of the throw sector, which the regulation tolerates to the extent of ± 0.001%, can cause variations of up to ± 8 cm for throws of 80 m (Mizera and Horvath, 2002; Hunter, 2005).

Jermy et al. (2014) reported the variations in aerodynamic resistance due to the different positions that the cable and the handle of the hammer can assume in the air after the hammer has been thrown. They noted that, while for relatively modest throws air resistance is mainly due to the motion of the hammer head, for longer ones the cable and the handle also influence the length of the throw. The length is also influenced by differences in the diameter of the hammer head: Dapena and Teves (1982) calculated a difference of about 30 cm on an 80 m throw made with a 120 cm diameter hammer compared to the same throw made with a 110 cm diameter hammer, which is the minimum allowed by the regulation.

All the studies on the ballistic and environmental aspects of the hammer throw provide a clear picture of the influence of these aspects on the length of the throw. However, a number of limitations must be acknowledged. First of all, the majority of the works reported results that are compared starting from the official measurements of the competition judges, which consider the distance from the landing point of the hammer to a measurement point that is different from the point of release of the hammer. Second, when calculating the throw variables, reference is usually made to a two-dimensional perspective on the sagittal plane, and often information on the horizontal plane is not included: for example, when evaluating the instant of release, the release height (the position on the Z axis) is taken into account, but in an overhead view it would also be possible to see the positions on the horizontal plane (i.e., X and Y axes), which affect the length of the throw. Finally, with reference to the environmental aspects, there are no studies that investigate the influence of air resistance when the hammer is accelerated by the thrower (Dapena and Teves, 1982; Goff, 2013).

## Kinematics of Center of Mass

There are three centers of mass that need to be considered when dealing with hammer throwing technique: the thrower's body center of mass (CoM_t_), the hammer's center of mass (CoM_h_) and the hammer-thrower system's center of mass (CoM_s_) (Dapena, 1986; Karalis, 1991). For each center of mass, the horizontal and vertical components of kinematic parameters (i.e., position and velocity, for example) must be considered Dapena (1986).

The position of the CoM_h_ is constant during the throw and is located near to the center of the hammer head for the male implement, and near the swivel for the female implement (Dapena and Teves, 1982). There are no specific studies regarding CoM_t_ and CoM_s_. Instant by instant the position of the CoM_t_ depends on the masses of the thrower's body segments and on their position/orientation, and this is valid also for the position of the CoM_s_ (Virmavirta and Isolehto, 2014).

In each of the phases of the throw, the centers of mass follow various displacements. There are no scientific studies on the center of mass motion during the preliminary rotations and release, whereas there are some publications on this subject during the turning phases (Dapena, 1986; Murofushi et al., 2007).

During the revolutions, the movement of the centers of mass can be broken down into their vertical and horizontal components. According to Murofushi et al. (2007) and Ohta et al. (2010) the horizontal component has a lower influence on the throw length than the vertical one. The vertical component shows a cyclical oscillation at each revolution for all three centers of mass (Dapena, 1984). However, their height does not vary simultaneously: in a frontal view of the hammer path there is an asynchrony between the highest point of the CoM_h_ with respect to that of CoM_t_. The CoM_s_ is located somewhere between the two, which have a phase lag between them of about 115°, but closer to the second. Furthermore, the lowest point of the CoM_s_ is located approximately in the middle of the DS phase, and the highest point approximately in the middle of the SS phase (Dapena, 1984, 1986). This is an important point not only from a biomechanical perspective but also from a technical point of view, since the thrower is searching for a dynamic balance of the throw by asynchronously opposing them self.

Murofushi et al. (2007) noted that for the Asian record holder of the hammer throw the lowest point of CoM_t_ is located after the beginning of the DS phase and coincides with the instant of the highest point of CoM_h_ of the hammer, while in athletes of minor qualification, these two centers of mass move much more in synchrony. Yuri Sedykh, during his world record throw, reached the lowest point of his center of mass immediately after the beginning of the DS phase (Otto, 1990). This study highlighted that lower-skilled throwers had a fairly synchronous rise of their hips with that of the hammer during the SS phase, unlike Sedykh, who had a phase delay of almost half a turn.

**Figure 3:**
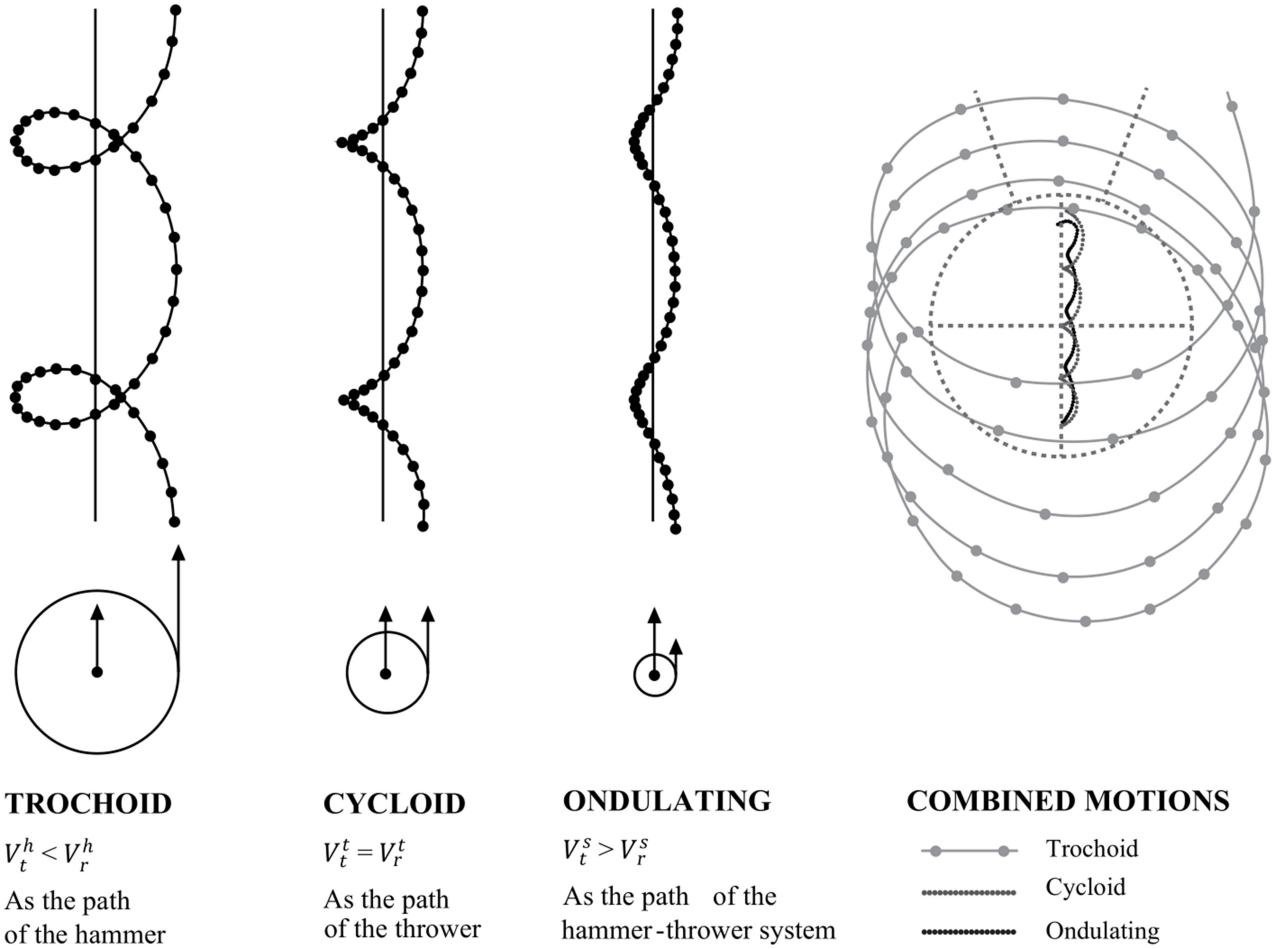
Rotation and translation in hammer throw. Adapted from Dapena (1986).

When analyzing the horizontal component of the centers of mass, it is crucial to consider that the throw consists of a movement composed of a translation and a rotation. In fact, the thrower while turning on itself gradually moves from the rear edge of the circle of throw to the front one, in the direction of throw. By defining $V_{t}^{h}$ the linear velocity relative to the translation and $V_{r}^{h}$ the linear velocity relative to the rotation, it has been reported that, for CoM_h_, $V_{t}^{h}$is $>V_{r}^{h}$, and its trajectory has a trochoid shape (Dapena, 1986). On the other hand, $V_{t}^{h}$ is approximately equal to $V_{r}^{h}$ for CoM_t_ which has a cycloid trajectory. Lastly, $V_{r}^{h}$is lower than $V_{t}^{h}$for CoM_s_ which shows a rather wavy shape, although with some differences between throwers (Figure 3). In fact, whereas for the CoM_t_ and CoM_h_ the trajectories are always cycloid and trochoid respectively, for the CoM_s_ there are two different types of trajectories (Dapena, 1984): one, called “loop pattern”, representing a trochoid/cycloid trajectory, although less accentuated than the trajectory described for the CoM_h_; a second, called “fixed point pattern”, in which the CoM_s_ has a rather straight horizontal movement (Dapena, 1986).

## Hammer's Path

When throws are analyzed in athletics a clear distinction immediately emerges between hammer throw and the other three disciplines. In shot put, discus throw, and javelin throw the center of mass of the implement is very close to the center of mass of the thrower's hand, whereas in hammer throw it is placed about 1.20 m away. This aspect has a strong impact on the biomechanical characteristics of the throwing technique and emphasizes the importance of analyzing the path of the hammer to fully understand the discipline. It has already been pointed out how, among several technical elements which the thrower must pay attention to, one of the most important is the maximization of the release velocity of the implement (Bartonietz, 2008). More specifically, it is crucial to maximize the tangential velocity at the instant of release, when the hammer interrupts its orbital trajectory, because it is bound to the hands of the thrower, and begins a parabolic trajectory (Mizera and Horvath, 2002).

### Relationship Between Tangential Velocity, Angular Velocity and Radius of Instantaneous Rotation

**Figure 4:**
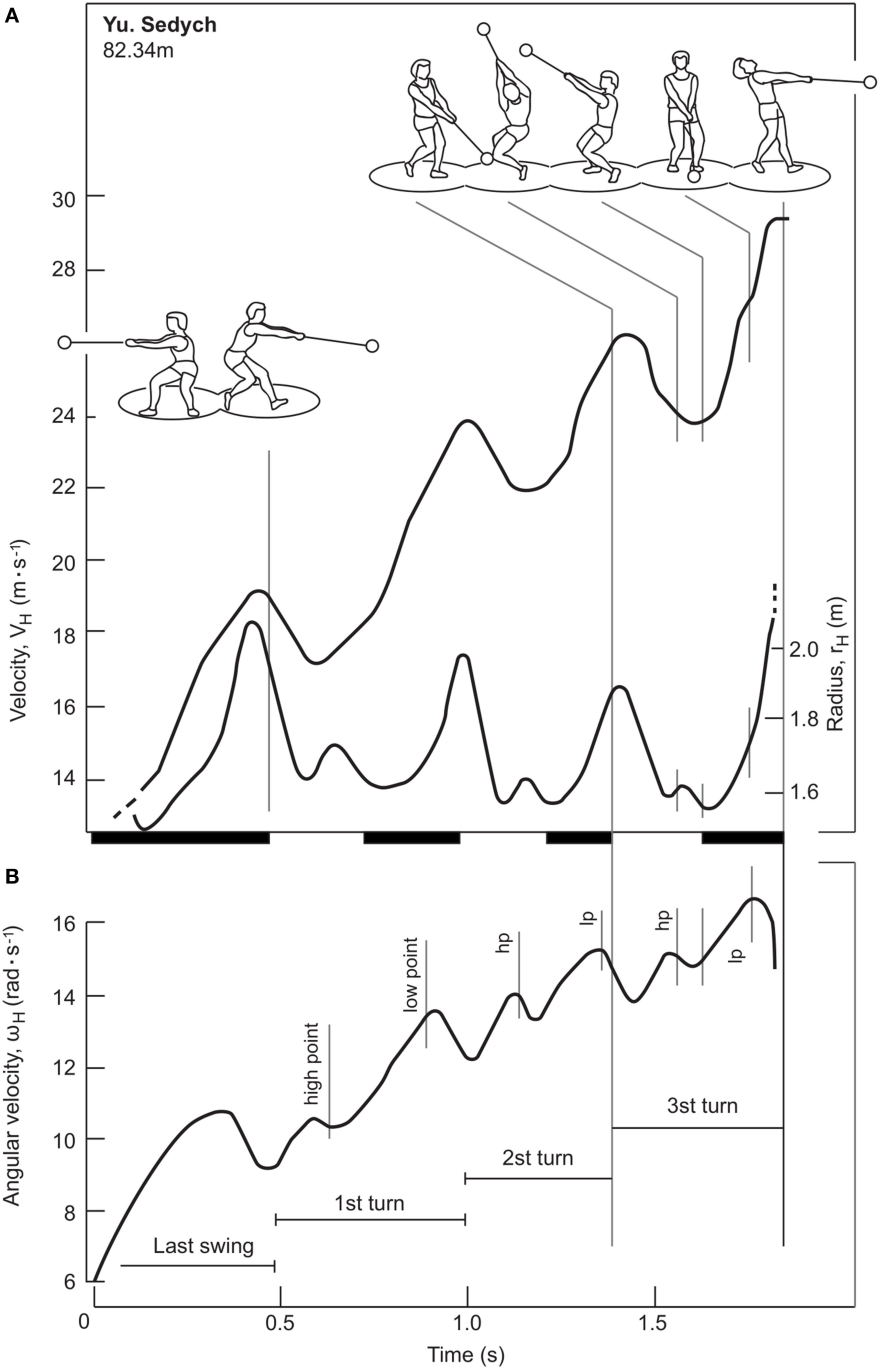
Adapted from Bartonietz (2008). Tangential velocity, V_H_, radius, r_H_, **(A)** and angular velocity, ω_H_ **(B)**. The bar on the x-axis shows the instants of the DS (black) and SS (white) phases.

To understand the development of the hammer head velocity during the throw, three parameters must be taken into account: the tangential velocity, the angular velocity and the radius of instantaneous rotation. These three parameters are related to each other and, in a simplistic and planar model of a material point (Brice, 2014), at any instant the linear velocity of the CoM_h_, **v**, is equal to:


(2) $$\begin{array}{l} \text{v}=\;r\cdot \omega \end{array}$$

where *r* is the radius of rotation (i.e., the distance of the point from the axis of rotation) and ω the hammer angular velocity. According to this relationship, therefore, it is possible to increase the linear velocity of a rotating point either by increasing the angular velocity or by increasing the radius. Thus, the thrower seeks the most efficient compromise between r and ω, in order to maximize v (Bartonietz, 2008; Brice, 2014). Typical trends of tangential velocity, angular velocity and instantaneous rotation radius during throw are shown in Figure 4.

### Tangential Velocity

Figure 4A shows the tangential velocity of the hammer (V_H_), from the last wind to the moment of release. The graph shows that during the last wind, which corresponds to the DS phase, the hammer linear velocity increases until the thrower lifts the right foot from the circle of throw. Subsequently, in each turn, the thrower alternates SS phases, in which the tangential velocity decreases, and DS phases, in which it increases, as indicated in several studies (Dapena, 1984; Susanka, 1986; Otto, 1990; Murofushi et al., 2005; Bartonietz, 2008; Brice et al., 2008; Rojas-Ruiz and Dávila, 2009; Lapp, 2014). It was also highlighted that the velocity increase during the DS phases and the velocity decrease during the SS phases are more prominent in the highly qualified athletes (Murofushi et al., 2005, 2007; Brice et al., 2008). Finally, after the last SS phase, the thrower releases the implement while the linear velocity of the hammer suddenly increases. The hammer velocity increases in each turn and reaches its highest value at the instant of final release (Dapena, 1984; Murofushi et al., 2005).

Several authors have argued that there is a cause-effect relationship between the increase in linear velocity during the DS phases and the decrease in linear velocity occurring in the SS phases (Otto, 1990; Bartonietz, 2008; Lapp, 2014). Other investigators have instead questioned this view, underlining how the increase in velocity and the DS phases are simultaneous, but are not necessarily related to each other (Dapena, 1984, 1986; Murofushi et al., 2005; Rojas-Ruiz and Dávila, 2009). In particular, the tangential velocity of the hammer seems to increase from the highest point (HP) to the lowest point (LP) and to decrease from the LP to the HP (Murofushi et al., 2005, 2007; Ohta et al., 2010; Brice et al., 2011, 2015). Therefore, it would appear that there is a partial overlapping in the velocity variations of the DS phase and the SS phase. Dapena (1984) also highlighted the influence of weight on velocity oscillations: by subtracting the vertical velocity component due to weight from the tangential velocity, a theoretical curve with less fluctuations, which in some cases are almost absent, is obtained. Furthermore, Rojas-Ruiz and Dávila (2009) indicated a decrease in the space traveled by the hammer along its path during the DS phase in the last two turns, compared to the first two, when the hammer reaches the highest velocity. If the cause-effect relationship between the length of the DS phase and the increase in the tangential velocity of the hammer is controversial, then the need to pursue a greater amplitude of the DS phase is also questionable (Murofushi et al., 2005; Rojas-Ruiz and Dávila, 2009).

Many authors did not consider the SS phase as a passive and preparatory phase for the subsequent DS phase, in which the possibility of accelerating the hammer is in any case greater. The velocity, due to the effect of weight (Dapena, 1984) and to the friction of the circle of throw with the left foot, decreases but the system can still benefit from a torque that accelerates the hammer, as the vertical reaction force of the foot left does not go through the CoM_t_ since it its lever arm differs from zero (Pozzo, 1992; Bartonietz, 2008).

### The Radius of Instantaneous Rotation and the Angular Velocity

As anticipated, the linear velocity represents the product of the angular velocity and the radius of rotation for every instant of the throw (Bartonietz, 2008). As the radius of instantaneous rotation (the distance of the CoM_h_ from the axis of rotation of the hammer-thrower system) increases, the angular velocity decreases and vice versa. Dapena et al. (2003) observed that the length of the radius also depends on the position of the CoM_h_ of the hammer. In addition, Dyson (1973) suggested that the radius depends on the mass of the thrower too. Finally, Dapena (1986) stated that the posture of the thrower represents the factor that has the greatest influence in determining the radius of instant rotation.

While the literature has unanimously identified the phases of the throw in which the linear velocity increases or decreases, the trends of radius of instant rotation are controversial. In particular, the studies by Bartonietz (2008) and Lee et al. (2000) identified in the DS phase the instants in which the instantaneous rotation radius increases, although they found that the maximum radius peaks at different instants: in the LP phase (Lee et al., 2000), and at the end of the DS phase Bartonietz (2008). In contrast, other authors indicated that the radius increases during the SS phase (Dapena and Feltner, 1989; Maronski, 1991; Murofushi et al., 2005; Ohta et al., 2010). Finally, Fujii et al. (2007) found that the radius mainly decreases during the SS phase, but, at the same time, reported an increase immediately after the hammer has reached the LP, in the DS phase. Measuring the radius of instantaneous rotation, which implies the definition of the axis of instantaneous rotation, has proven considerably critical in previous investigation due to the difficulties of the measurement itself as well as the limited number of participants evaluated in most studies (Murofushi et al., 2005, 2007).

During the throw, angular velocity tends to increase, together with the linear velocity, while the radius slightly decreases. However, even considering this trend, Dapena and Feltner (1989) and Maronski (1991) pointed out that the throwers, besides looking for the best compromise between angular velocity and radius of instant rotation, seek a rapid shortening at the instant of final release (Dapena and Feltner, 1989; Maronski, 1991; Bartonietz, 2008).

With regard to the strategies to maximize performance, on one side Maronski (1991) highlighted that there is an advantage for the thrower to maintain a radius length as constant as possible during the turns, except for the release phase. On the other side, Dapena and Feltner (1989) described the thrower's posture by identifying two different technical variants: one named “countering with the shoulders technique” and the other “countering with the hips technique”. The first consists of keeping a position and a stable posture in opposition to the hammer during the revolutions. The second involves a backward movement of the hips while the shoulders come forward to achieve the longest possible path of the hammer. This type of technique allows, for a given tangential velocity of the hammer, to have a greater radius of rotation and a lower angular velocity. Since the thrower's rotation velocity on itself is lower than in the first technique, the “countering with the hips technique” allows for greater effectiveness in producing force. This is due to the possibility for the muscles involved to contract across a longer period of time (Hill, 1922; Dapena and Mc Donald, 1989). Therefore, this additional force leads to a greater angular momentum. For the “countering with the shoulder technique” the tendency is to maintain such a position for the entire throw, while for the “countering with the hips technique” the tendency is to pull back the shoulders and bring the hips forward during the throw, after the first two turns, for athletes who use a four-turns technique. This causes the radius to gradually decrease while improving the angular velocity of the thrower. It has been argued that the factors precluding maximization of the radius, as a consequence of juxtaposition of the hips, are an excessive load on the spine and an insufficient strength in the shoulder musculature.

### Translation, Inclination, and Torsion of the Path

**Figure 5:**
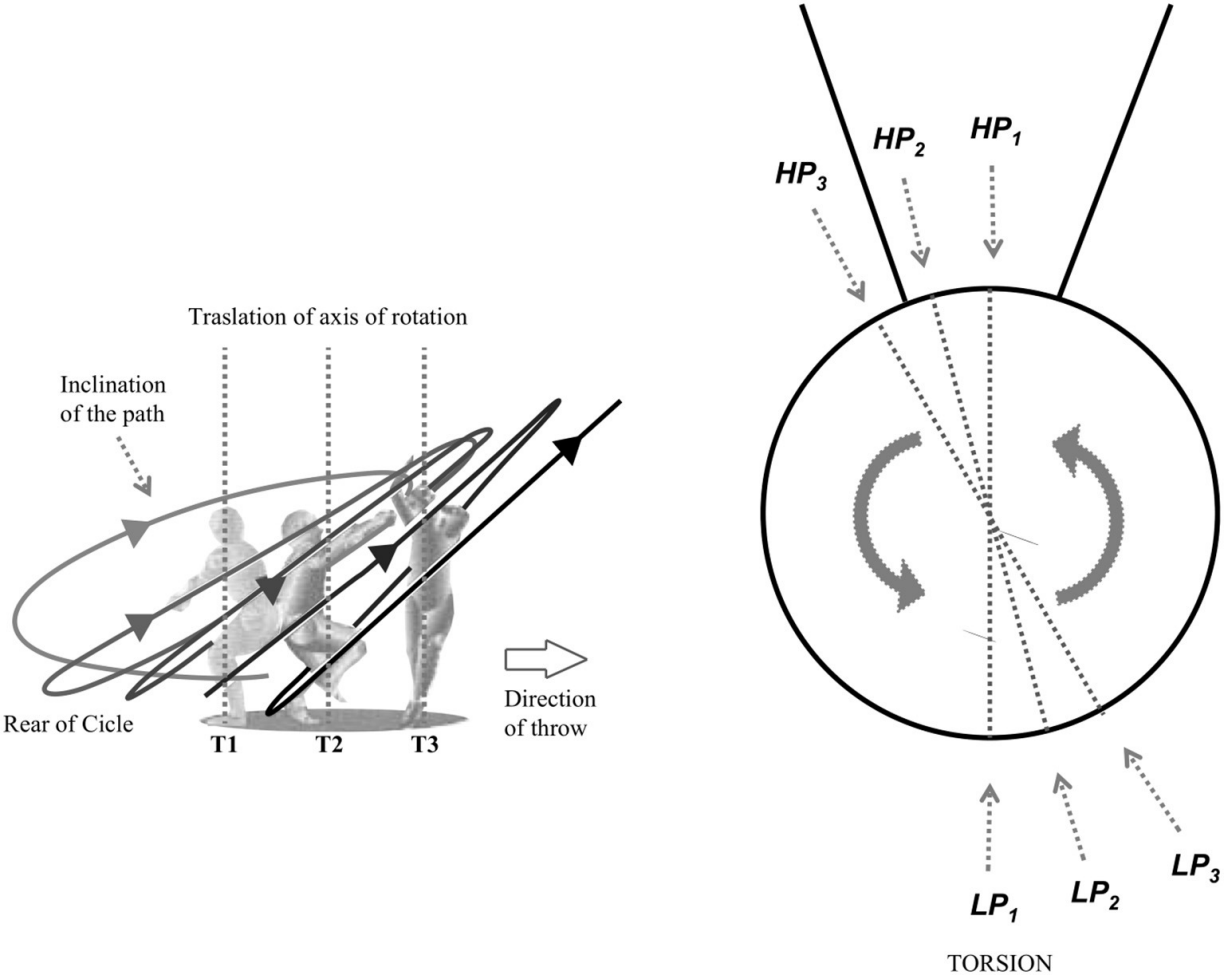
Inclination, translation and torsion.

Translation of the path consists in the movement along the circle of the axis of instantaneous rotation of the hammer's path caused by the displacement of the thrower during the throw (Figure 5). This occurs mainly in the direction of throw, that is, from the rear of the circle to the front. In fact, as previously reported, the movement of the hammer-thrower system is the result of a rotation and a translation. In particular, the displacement is mainly caused by the heel-toe footwork during the turning phase, that goes approximately from 90 ° to 180 ° azimuth, i.e., in the first half of the SS phase of each turn (Pozzo, 1992). It is important to note that this translation affects the tangential velocity of the hammer, because it adds a linear velocity, which is caused by the displacement of the hammer-thrower system and its center of mass along the circle. Furthermore, the change in velocity due to translation is not uniform, since the heel-toe passage of the left foot that makes the thrower advance across the circle occurs in a limited portion of the turn (Bondarchuck, 1987; Bartonietz, 2008).

Path inclination represents the angle between the horizontal and the straight line passing by the HP and LP that the hammer reaches in every turn, in a lateral view of the circle (Figure 5). The inclination generally increases during the turns, although with different patterns depending on the throwers. As inclination varies, so vary the different kinematic, dynamic and technical characteristics (respectively, the distance of the hammer from the axis of rotation on the horizontal plane, the influence of weight, and the posture of the thrower in various phases of the turn) (Bondarchuck, 1987; Pozzo, 1992; Bartonietz, 2008).

The path torsion, as described in the overhead view of Figure 5, represents the displacement of LP and HP reached by the hammer, turn by turn, within the orbit compared to the previous turn. In modern technique, throwers have a tendency to hold their LP in front of their shoulders when they are in the middle of the DS phase. In the past, the path of the hammer reached its LP approximately in front of the right foot (Bondarchuck, 1987). In addition, a gradual and slight shift to the left of LP and HP during the turns is usually observed in many throwers. As the position of LP and HP changes from turn to turn, the thrower's posture also changes during the throw and the tensions on the cable of the hammer. Furthermore, changes in LP and HP positions influence the hammer release height (Bartonietz, 2008). In the scientific literature there is no study addressing the issue of path torsion and describing the position of LP and HP in the frontal plane.

### The Path of the Hammer in Winds

As previously described, preliminary winds represent those rotations performed by the thrower before the beginning of the turns in order to overcome the inertia of the hammer and thus favoring the successive phases of the throw. With reference to the winds, Dapena and Feltner (1989) reports that the inclination of their path increases from turn to turn, as LP reaches a lower altitude with each wind, and HP reaches higher altitudes. This has been demonstrated by Rozhkov et al. (2020), who carried out an analysis on the main kinematic parameters of the winds among the finalists of the London World Championships in 2017. At the end of the winds, a phase delay of about 180° was found between the thrower and the hammer, which remained rather constant during the turns (Rozhkov et al., 2020). In addition, winds considerably vary among athletes, who look for both efficient and comfortable movement patterns. Although technical articles underlined the importance of the winds and how an effective execution of the winds is crucial for a successful throw (Bondarchuck, 1987; Dapena and Feltner, 1989; Pozzo, 1992; Bartonietz, 2008), the scientific literature focusing on this subject is scarce. Further investigations regarding the kinematics of the winds are thus required.

## Separation Angle Between Pelvis and Thorax

**Figure 6:**
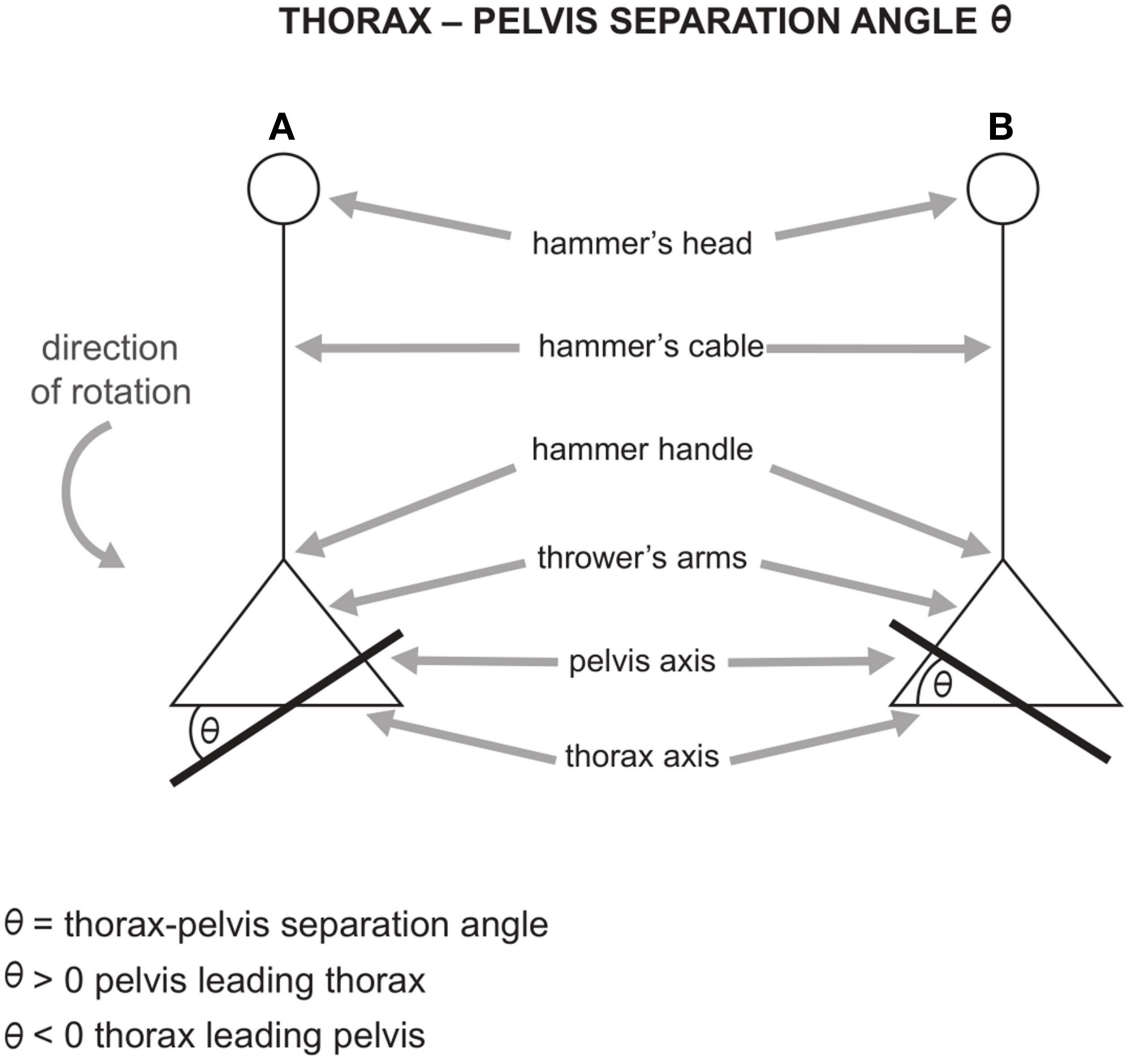
**(A)** positive separation angle (pelvis leading thorax) and **(B)** negative separation angle (thorax leading pelvis). Adapted from Brice et al. (2018).

One of the most important elements of the hammer throw technique is the angle of separation between the axis of the pelvis and the axis of the thorax (Figure 6). Although the importance of this parameter is well recognized in various technical articles, there are very few scientific works on the subject. The angle of separation between the axis of the pelvis and the axis of the thorax θ is considered as positive when, in a counter clockwise rotation (in a right-handed athlete), the axis of pelvis has a value in azimuth angles greater than that of the thorax, namely when the pelvis guides and anticipates the thorax. Conversely, θ is negative when the axis of the thorax has a greater value than that of the pelvis. In this case, the thorax guides and anticipates the pelvis. During the turns, throwers usually have a positive separation angle. Furthermore, the tendency of throwers, including that of the world leader Yuri Sedykh, is to reduce the separation angle in the DS phase during the turns and to increase it during the SS phase (Bartonietz, 2008; Brice et al., 2018; Sedykh and Strelnitski, 2018).

Bartonietz (2008) highlighted that the increase of θ in the SS phase has a positive impact on the throw only if the forward movement of the pelvis on the thorax is reduced in the DS phase. In fact, maintaining a high value of θ during both phases of the turns causes a decrease in the radius of rotation of the system. Brice et al. (2018) pointed out that there is a high positive correlation between the length of the throw and the decrease of θ in the DS phase of the first two turns. The authors argued that reducing θ by as much as possible during DS, particularly in the first two turns, could allow greater throwing distance. They suggested that throwers should strongly focus on the reduction of θ. since rotation speed is particularly low in turns 1 and 2, likely making a technique alteration more achievable.

Although increasing the separation angle in the SS phase and decreasing it in the DS phase contribute to an increase in tangential velocity of the hammer during the turns, this should not lead to an excessive value of θ in the SS phase (Bondarchuck, 1987; Bartonietz, 2008; Brice et al., 2008). If this occurs, there are two consequences: first, an excessive decrease of the radius could lead to a substantial decrease in tangential velocity of the hammer (see Eq. 1.2); second, the thrower could find themself in a more unstable position, since in the SS phase of each turn he/she is in contact with the ground only with one foot. This instability would not allow the thrower to maximize the hammer acceleration. Moreover, as previously stated, decreasing the separation angle θ is a fundamental condition for a good execution of the throw. Bartonietz (2008) showed that during the turns the greatest values of separation angle θ are reached in the second part of the SS phases of each turn. Additionally, the authors reported that the optimal values of θ oscillate between 20° and 40° during this specific phase.

The scientific literature clearly indicates that the strategy to increase tangential velocity of the hammer is to increase the angle of separation between the pelvis and the thorax in the SS phase, and decrease the separation angle in the DS phase, especially in the first two turns, to increase the tangential velocity of the hammer (Murofushi et al., 2007; Bartonietz, 2008; Brice et al., 2018). However, in the SS phase, the magnitude of the angle of separation between the pelvis and the thorax must be contained, in order not to compromise the tangential velocity of the hammer and the dynamic balance of movement.

After examining the role of the angle of separation during the turns, some further considerations on this technical aspect must be made during the winds and the final release. Since there is need to create a positive separation angle θ from the beginning of the turns, the technical literature pointed out that the main objective of the winds is to create a positive θ before moving to the turning phase. Since the hammer rotates around the thrower, who is placed on the ground, the separation angle θ assumes positive and negative values in each turn. At the end of the winds, θ must be positive and the thrower should achieve the optimal separation angle. This is not a simple task because, during the winds of the hammer, the thrower is stationary with his feet, while during the turns the thrower rotates with their feet together with the hammer. Therefore, moving from winds to turns, which in the technical literature is called “entry to the first turn”, is considered as a delicate and crucial phase for the success of the throw (Bondarchuck, 1987; Pozzo, 1992; Bartonietz, 2008).

In the final release phase, θ becomes negative, since the feet are placed on the floor and the trunk continues its rotation to accelerate the hammer as much as possible until the instant of the final release is reached, at about 90° azimuth (Susanka, 1986; Murofushi et al., 2007; Brice et al., 2008).

## Forces Involved in the Throw

Three groups of forces must be considered in the hammer throw movement according to the system under analysis: the forces acting on the hammer, on the thrower and on the entire hammer-thrower system (Brice, 2014).

### Forces Acting on the Hammer

The forces acting on the hammer are the environmental forces, weight and the force applied by the thrower to the hammer through the hammer cable. The environmental forces acting on the hammer can be analyzed before and after the hammer is released from the thrower. The latter have already been discussed by analyzing the ballistic aspects of the throw, while there are no scientific studies on the environmental forces acting on the hammer before it is released (Dapena, 1984). Considering the other two mentioned forces, the cable tension can be analyzed, instant by instant, using a reference frame having the center of mass of the hammer as its origin, in the tangential, radial and normal directions to the plane of the trajectory. The effect on the hammer tangential velocity of the normal components of the forces is negligible whereas the resultant force along the radial direction affects the radius of curvature of the implement during the revolutions. The only components of the forces acting on the hammer that influence the tangential velocity are the tangential ones (Dapena, 1984; Pozzo, 1992; Brice, 2014).

**Figure 7:**
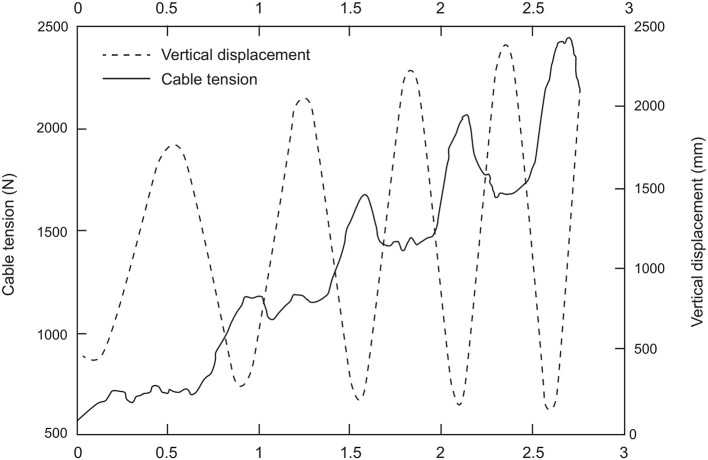
Adapted from Brice et al. (2008). Development of the cable tension during the hammer throw.

**Figure 8:**
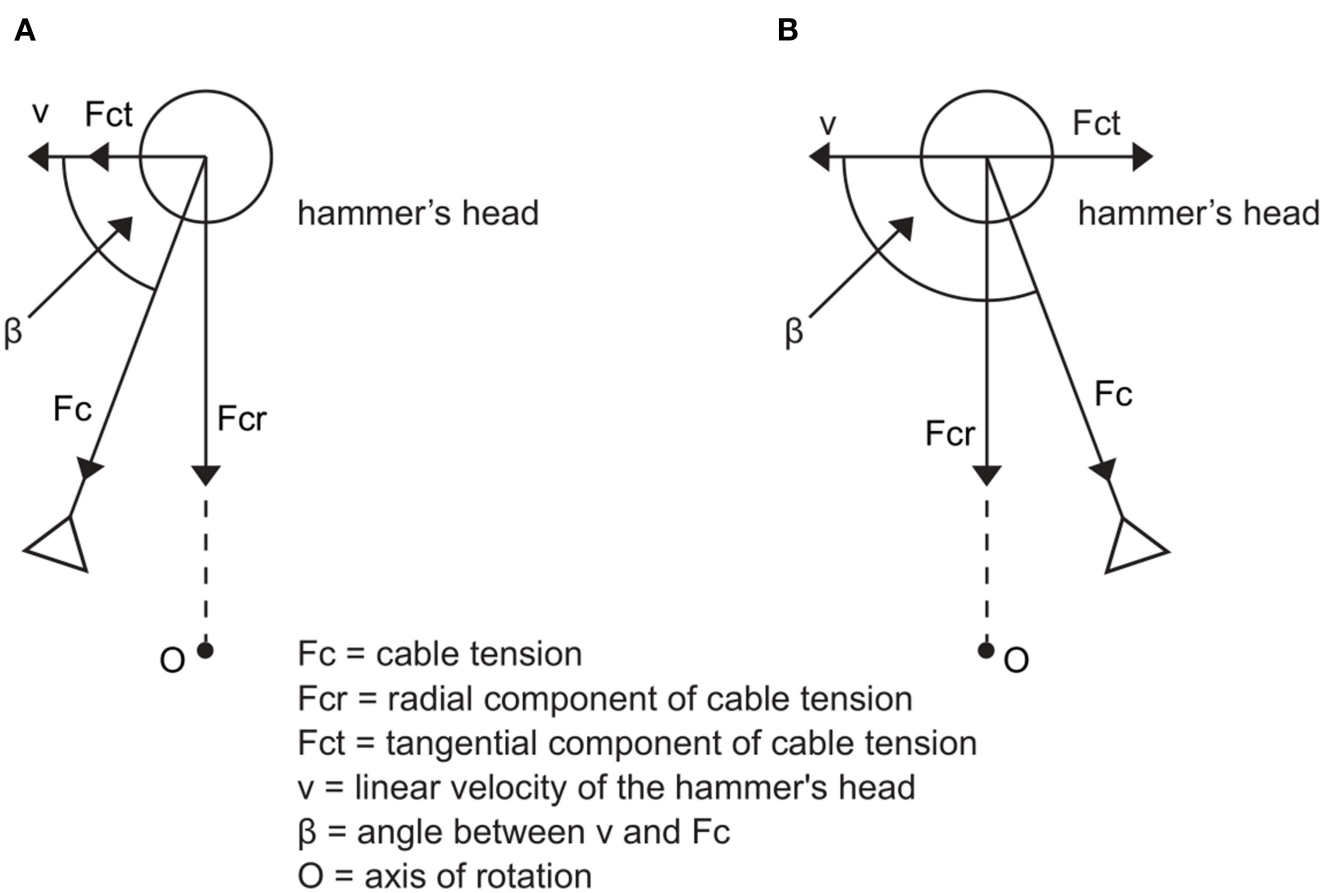
Components of cable tension when the cable tension vector is pulling in front **(A)** and behind **(B)** the axis of rotation. Adapted from Brice et al. (2011).

The motion law of the resultant force applied to the hammer has a development similar to the tangential velocity: the period of fluctuations is one turn, the phases of increase in the applied force and tangential velocity are found in each DS (while decrements are observed in the SS phases) and the peaks of maximum cable tension and hammer acceleration lie near the LP of hammer path (Figure 7). The development of the force curve, therefore, suggests that throwers apply a force from the HP to the LP of the turns, using the acceleration determined by the weight of the hammer. However, previous studies did not show a causal link between the forces applied by the thrower and the velocity increase. Dapena and Feltner (1989) separated the effect of the accelerations caused by weight and the translation across the circle of throw from the effects caused by the rotation. In particular, Brice et al. (2011) found that the horizontal component of the hammer tangential velocity increases if the force is exerted in front of the axis of instantaneous rotation (β <90° in Figure 8A), and a negative variation if the force is applied behind (β > 90° in Figure 8B). In both cases, the normal component of the cable tension acts perpendicularly to the plane of rotation and points upwards, while the normal component of weight points downwards (Murofushi et al., 2005, 2007; Brice et al., 2008; Gesser et al., 2021). In addition, Brice et al. (2011) analyzed the impact of a negative tangential component of the cable tension on the development of hammer velocity. The authors suggested that throwers should reduce β as much as possible when the tangential component of cable tension is negative (Brice et al., 2011).

The main factors affecting the amplitude of the force, aside from gender, are the technical ability of the thrower and their ability to apply force. With respect to the last two factors, Dapena and Feltner (1989) pointed out that the former (technical ability of the thrower) is a prerequisite for effectively expressing the latter (ability to apply force). In this respect, Bartonietz (2008) highlighted that the role of the leg muscles differs from the role of trunk and arm muscles. In fact, leg muscles are major contributors to generating force whereas trunk and arm muscles are crucial to transfer force to the hammer. There are only few works concerning the relationship between strength and force in throwing disciplines. A few authors have identified some of the key factors influencing force magnitude in the thrower. Among these, lean mass, muscle fiber type and architectural characteristics of the muscles appear to predominantly contribute (Zaras et al., 2021). Mass significantly influences the amount of force produced because heavier throwers usually also have a greater volume of muscle allowing for an enhanced capacity to apply forces on the hammer cable (Billeter et al., 2003; Gutierrez-Davila and Rojas-Ruiz, 2005; Okamoto et al., 2007; Singh et al., 2011). Furthermore, the greater the mass of the thrower, the greater the radius of instant rotation of the hammer (Dyson, 1973). The radius passes through the feet of the thrower and the instantaneous rotation center of the hammer-thrower system depends on the mass and on the spatial orientation of the hammer and the thrower (Dapena and Feltner, 1989). With regard to muscle contractile characteristics, physiological studies reported that throwers tend to have a volume of fast-twitch muscle fibers relatively higher than the sedentary population or non-competitive amateur athletes (Billeter et al., 2003; Terzis et al., 2009, 2010). It was also observed that both the percentage of fast-twitch fibers and their volume, which are greatly influenced by genetic factors, can increase over the years with specific training for power sports. It was also noted that this phenomenon is reversible when competitive activity is interrupted (Billeter et al., 2003).

### Forces Acting on the Thrower

There are three types of forces that act on the thrower: the overall weight, the reaction force exerted by the ground (ground reaction force–GRF) and the reaction force to the cable tension, which is equal and opposite to the latter. In particular, weight has only a vertical component, while the other two types of force have both a vertical and a horizontal component (Dapena, 1986; Dapena and Feltner, 1989). There are several scientific studies that directly investigate how the reaction force to the cable tension acts on the thrower (Wang et al., 2014, 2018a,b; Wan et al., 2020), but very few that deal with the GRFs (Murofushi et al., 2005, 2007). This is probably due to the difficulty in carrying out these measurements. Some authors indicated that, during the throw, the reaction force to the cable tension increases from turn to turn, while the GRFs increase much less, with the exception of the final release phase (Murofushi et al., 2005; Brice et al., 2008; Brice, 2014). Murofushi et al. (2007) also found that in elite athletes there is a greater increase in the reaction force to the cable tension than in lower-ranked athletes. By analyzing the vertical components of the forces acting on the thrower, a positive acceleration is observed from the LP to the HP of CoM_t_, which in the turns is particularly evident in the DS phases. In these phases, during which the thrower has both feet in contact with the ground, the thrower is characterized by a greater stability with respect to the SS phases and, thus, he/she can exert greater forces (Murofushi et al., 2007). The positive acceleration is due to the fact that the sum of the vertical components of the GRFs is greater than the weight of the thrower. The opposite phenomenon is observed when the sum of the two types of force is lower than the force of gravity, i.e., mainly in the phases of SS (Dapena, 1986).

The horizontal component of the reaction force to the cable tension has a much greater magnitude than the horizontal component of the GRFs, while that of gravity is equal to zero (Dapena, 1986). Therefore, there is a horizontal resultant force acting on the thrower in the direction of the reaction force to the cable tension. This force provides centripetal acceleration that allows the thrower to rotate around the CoM_s_ (Dapena, 1986).

### Forces Acting on the Hammer-Thrower System

When the hammer-thrower system is analyzed, the forces exchanged between the thrower and the hammer are not taken into account, and the movement of the CoM_s_ is influenced by weight and the GRF (Dapena, 1986). As described for the vertical components of the forces that accelerate the CoM_t_, a positive acceleration is also transmitted to the CoM_s_ when the GRF is greater than the weight of the hammer-thrower system. This occurs mainly in the DS phase, during which the CoM_s_ passes from the LP to the HP. On the opposite, when the vertical GRF is lower than the weight of the system, mainly in the SS phases, the CoM_s_ accelerates downwards and moves from the HP to the LP. These oscillations of the CoM_s_, with positive accelerations in the DS phases and negative accelerations in the SS phases, have been observed both in elite throwers and in lower-ranked athletes (Dapena, 1986). That being said, Murofushi et al. (2007) found important differences in the vertical components of GRF between athletes of different levels. In elite throwers, who show similar levels of strength in the right and left foot, the right foot shows a very high peak of ground impact force at each turn, when the DS phase begins. Furthermore, during the DS phase of each turn, the GRF shifts gradually from the right foot to the left foot. In athletes with a lower performance level, these phenomena are much less noticeable (Dapena, 1986; Murofushi et al., 2007).

The only horizontal force that influences the movement of the CoM_s_ is the horizontal component of the GRF. Dapena (1986) observed that the horizontal movement of the CoM_s_ is rather straight across the diameter of the circle, or slightly cycloid, unlike the vertical movement, which has much greater oscillations. Thus, the horizontal component of the GRF is small compared to the vertical one. Murofushi et al. (2007) confirmed Dapena's hypothesis in a study in which he directly measured the horizontal and vertical components of the GRF.

## Conclusions

This review focuses on the biomechanics of hammer throw. All the articles published in indexed scientific journals and the main technical articles dealing with the analysis of the hammer throw technique from a biomechanical perspective were analyzed and their results summarized and discussed. A subdivision of the topics by thematic areas and their critical exposition was made. In particular, the ballistic and environmental aspects of the discipline and the motion of the centers of mass of the hammer-thrower system were described; the movement of the hammer in its characteristic path was also analyzed and the forces involved in the throw described. The analysis of these elements revealed that the modern throwing technique has many elements in common with that used by the best throwers in the Eighties and, for this reason, many articles in the scientific literature are still up to date. In addition, our work highlights that improving the understanding of the athletes' throws requires the evaluation of the whole hammer-thrower system rather than the two subsystems (hammer and thrower) alone. The optimal technique involves applying forces to maximize the throwing performance which depends, besides ballistic and environmental aspects, on achieving the highest linear velocity of the hammer during the acceleration phase. Thus, the present work indicates that the main factors influencing the throwing technique are related to the centers of mass of the three systems, the amplitude and inclination of the hammer's orbit, the duration of the DS and SS phases in every turn and the variation of the angle θ throughout the different phases of the throw. Nonetheless, new technologies would allow re-examinations of various aspects of the throw and, in this regard, wearable technology certainly represents the new frontier in research (Ohta et al., 2008; Kelley, 2014; Wang et al., 2016). Moreover, a comprehensive analysis of some ballistic and environmental aspects is still needed. In particular, both preliminary winds and entry to the first turn have not been assessed in the scientific works, which lack an accurate description of the biomechanical characteristics of these two phases. In addition, performing an adequate biomechanical evaluation of hammer throw requires the definition of the rotation axes involved in the throw, given the poor relevance that has been attributed to this aspect so far.

## Author Contributions

GC, RB, and AM conceived and designed the literature review. GC conducted the literature screening, data extraction, and created the figures. GC, RB, EB, VC, GV, and AM participated in drafting and revising the manuscript. All authors contributed to the article and approved the submitted version.

## Conflict of Interest

The authors declare that the research was conducted in the absence of any commercial or financial relationships that could be construed as a potential conflict of interest.

## Publisher's Note

All claims expressed in this article are solely those of the authors and do not necessarily represent those of their affiliated organizations, or those of the publisher, the editors and the reviewers. Any product that may be evaluated in this article, or claim that may be made by its manufacturer, is not guaranteed or endorsed by the publisher.
